# Miniaturized Bioaffinity Assessment Coupled to Mass Spectrometry for Guided Purification of Bioactives from Toad and Cone Snail

**DOI:** 10.3390/biology3010139

**Published:** 2014-02-13

**Authors:** Ferry Heus, Reka A. Otvos, Ruud L. E. G. Aspers, Rene van Elk, Jenny I. Halff, Andreas W. Ehlers, Sébastien Dutertre, Richard J. Lewis, Sybren Wijmenga, August B. Smit, Wilfried M. A. Niessen, Jeroen Kool

**Affiliations:** 1 AIMMS Division of BioMolecular Analysis, Faculty of Sciences, VU University Amsterdam, De Boelelaan 1081, 1083 HV Amsterdam, The Netherlands; E-Mails: ferryheus@gmail.com (F.H.); r.a.otvos@vu.nl (R.A.O.); j.i.halff@student.vu.nl (J.I.H.); w.m.a.niessen@vu.nl (W.M.A.N.); 2 Department of Molecular and Cellular Neurobiology, Center for Neurogenomics and Cognitive Research, Neuroscience Campus Amsterdam, VU University Amsterdam, De Boelelaan 1085, 1081 HV Amsterdam, The Netherlands; E-Mails: rene.van.elk@vu.nl (R.E.); guus.smit@vu.nl (A.B.S.); 3 Department of Biophysical Chemistry, Institute for Molecules and Materials, Radboud University Nijmegen, Heyendaalseweg 135, 6525 AJ Nijmegen, The Netherlands; E-Mails: r.aspers@nmr.ru.nl (R.L.E.G.A.); s.wijmenga@nmr.ru.nl (S.W.); 4 AIMMS Division of Organic Chemistry, Faculty of Sciences, VU University Amsterdam, De Boelelaan 1081, 1083 HV Amsterdam, The Netherlands; E-Mail: a.w.ehlers@vu.nl; 5 The Institute for Molecular Bioscience, The University of Queensland, St Lucia, Queensland 4072, Australia; E-Mails: s.dutertre@imb.uq.edu.au (S.D.); r.lewis@imb.uq.edu.au (R.J.L.); 6 hyphen MassSpec Consultancy, de Wetstraat 8, 2332 XT Leiden, The Netherlands

**Keywords:** on-line microfluidics, nano-liquid chromatography-mass spectrometry (nano-LC-MS), *Lymnaea stagnalis* acetylcholine binding protein (Ls-AChBP), *Conus textile*, *Bufo alvarius*, *Bufo marinus*

## Abstract

A nano-flow high-resolution screening platform, featuring a parallel chip-based microfluidic bioassay and mass spectrometry coupled to nano-liquid chromatography, was applied to screen animal venoms for nicotinic acetylcholine receptor like (nAChR) affinity by using the acetylcholine binding protein, a mimic of the nAChR. The potential of this microfluidic platform is demonstrated by profiling the *Conus textile* venom proteome, consisting of over 1,000 peptides. Within one analysis (<90 min, 500 ng venom injected), ligands are detected and identified. To show applicability for non-peptides, small molecular ligands such as steroidal ligands were identified in skin secretions from two toad species (*Bufo alvarius* and *Bufo marinus*). Bioactives from the toad samples were subsequently isolated by MS-guided fractionation. The fractions analyzed by NMR and a radioligand binding assay with α7-nAChR confirmed the identity and bioactivity of several new ligands.

## 1. Introduction

Besides being promising pharmacological tools, bioactive compounds from “natural sources” are considered of interest to the pharmaceutical industry as potential medicines [1]. Compounds from natural sources comprise an array of molecules, which have evolved to target specific biological tasks and aim at, e.g., avoidance of predation, immobilizing a prey, or preventing a prey’s reproduction. A challenge of modern drug discovery is to identify these compounds for affinity towards a biochemical process or a specific protein target, to isolate them and to develop such compounds into innovative drugs. The first step is the selection of a natural source, which could be an extract used for (traditional) medicinal purposes. Other sources rich in different biologically active compounds are animal venoms, which contain toxins, either to catch prey or to evade predation.

Cone snails, such as *Conus textile*, are predatory sea snails with venom containing more than a thousand conopeptides, which are small peptides with 12 to 35 amino acids and some disulfide bridges [2]. Many of these conopeptides target specific ion channels in the peripheral nervous system and are involved in the transmission of pain stimuli [3,4] often with high specificity and affinity, such as the nicotinic acetylcholine receptor nAChR [5,6,7], which therefore may be potential biopharmaceuticals. This is reflected by the recent FDA approval of the conopeptide-derived drug under the name Ziconotide (Prialt), which blocks a neuronal *N*-type voltage-gated calcium channel in the treatment of neuropathic pain [7,8]. The α9α10-type nAChR bioactive conopeptide ACV1 (from Metabolic Pharmaceuticals Limited, Melbourne, Australia), involved in pain relief, reached clinical trials, but was abandoned due to efficacy lack in humans [9]. Another conopeptide-based drug candidate, xen2174 (from Xenome Ltd., Brisbane, Australia), increases norepinephrine levels in the spinal cord. An inherent advantage of conopeptide analysis is that identification is relatively straightforward as it can be accomplished with MS^2^ peptide sequencing [10,11,12,13] (after reduction).

Toad skin excretions comprise a complex cocktail of small peptides, tryptamine derivatives (with masses between 100 and 400 Da) and steroidal compounds (with masses between 400 and 800 Da), and might be a natural source for bioactive compounds [14]. In contrast to conopeptides, they are expected to pass the blood brain barrier more easily. During evolution, toads produced the tryptamine derivatives from serotonin, which has been the archetype “scaffold” [15]. These compounds, being excreted from glands in the skin of these toads, serve to deter animals, function as anti-predation toxins, and are known to cause convulsion, hallucinogenic effects and (even) death [16]. Toad skin excretions from the Cane toad skin (*Bufo marinus*) and the Colorado River toad skin (*Bufo alvarius*)may also exhibit AChBP affinity.

As the compounds in natural extracts are present in complex mixtures, the screening of these extracts for bioaffinity and identification of the bioactives needs to be optimally combined. Commonly, separation is the first step in natural extract screening after which biochemical assays on collected fractions are applied to screen for bioaffinity. Typically, “hits” are validated biochemically and their structure is elucidated by mass spectrometry (MS) and nuclear magnetic resonance spectroscopy (NMR), after iterative purification rounds with orthogonal separations. This traditional approach is laborious and requires relatively large amounts of sample [5,17,18,19]. A specific challenge of natural extract screening is the accurate matching of bioactives with their chemical structure [20,21,22,23,24]. Traditionally, bioassays are performed after low-resolution fractionation of the extract. Besides being laborious, this process is often only capable of identifying a small portion of the bioactives present [25], and closely eluting bioactives are not screened individually.

New screening approaches for natural extracts, e.g., affinity-selection MS (AS-MS) [26], ultrafiltration MS [27] and high-resolution screening (HRS) [28,29,30,31,32], implement integrated liquid chromatographic (LC) separation and LC-MS. AS-MS techniques measure binding between a protein and its potential ligands by separating the protein-ligand complexes from the non-binding mixture components. Identification of the ligands is done subsequently with MS or MS-MS, whereas HRS techniques apply biochemical assays coupled on-line to LC-MS.

To screen for bioactive compounds that act on the membrane-bound nAChRs, the soluble homolog acetylcholine binding protein (AChBP) can be used [33,34,35,36]. Recently, a HRS system, featuring an on-line fluorescence enhancement biochemical assay, for AChBP screening was developed [37]. The assay format was subsequently miniaturized in order to enable screening of venoms of which only limited amounts are available for analysis [38]. The miniaturized format was successfully applied for profiling of neurotoxic snake venoms [39]. An advantage of this “on-line” HRS technique lies in the ability to directly pinpoint ligands, even when the compounds in the mixture are poorly separated, which is often the case when screening complex venom samples.

Here, we combine HRS, featuring nano-LC separation, on-line miniaturized biochemical assaying and parallel MS analysis for rapid ligand identification, to MS-guided purification to enable full structure elucidation by, e.g., NMR or advanced MS technologies, and biological assaying of the purified ligands towards the human α7-nAChR using a radioligand binding assay (RBA). Using this screening approach, we readily identified AChBP ligands in cone snail venom (*Conus textile*) and in natural extracts from Cane toad skin (*Bufo marinus*) and the Colorado River toad (*Bufo alvarius*)*.*

## 2. Experimental

### 2.1. Chemical and Biological Reagents

Ls-AChBP (from the snail species *Lymnaea stagnalis*) was expressed from *Baculovirus* using the pFastbac I vector in Sf9 insect cells and purified from the medium, as described by Celie *et al.* [35]. Human neuroblastoma cells (SH-SY5Y) expressing human R7 nAChRs were obtained from Christian Fuhrer (Department of Neurochemistry, Brain Research Institute, University of Zurich, Zurich, Switzerland). The fluorescent tracer ligand DAHBA, (*E*)-3-(3-(4-diethylamino-2-hydroxybenzylidene)-3,4,5,6-tetrahydropyridin-2-yl)pyridine, was synthesized in house [37]. Human neuroblastoma cells (SH-SY5Y) expressing human α7-nAChR were washed with PBS, collected as a pellet and stored at −80 °C until further use. ^3^H-Methyllycaconitine (^3^H-MLA) was from American Radiolabeled Chemicals Inc. (St. Louis, MO, USA). KCl, DL-dithiothreitol (DTT), guanidine-HCl, NaCl, trizma base, HCl, Tween 20, [Met^5^]-enkephalin and human angiotensin I, and *Bufo marinus* and *Bufo alvarius* extracts were purchased from Sigma-Aldrich (Zwijndrecht, The Netherlands). KH_2_PO_4_, Na_2_HPO_4_ and NH_4_HCO_3_ were obtained from Riedel-de-Haën (Seelze, Germany). I-Nicotine (99.0%) was purchased from Janssen Chimica (Beerse, Belgium). Enzyme linked immunosorbent assay (ELISA) blocking reagent (ELISA-BR) was purchased from Hoffmann-La-Roche (Mannheim, Germany). Mca-Lys-Pro-Leu-Gly-Leu-Dap(Dnp)-Ala-Arg-NH_2_ was from Bachem (Bubendorf, Switzerland). Methanol-d4 was purchased from Cambridge Isotopes Laboratories, Inc. (Buchem B.V., Apeldoorn, The Netherlands). ULC/MS grade trifluoroacetic acid (TFA; 99.95%), formic acid (FA; 99.95%) methanol (MeOH; 99.98%) and acetonitrile (ACN; 99.97%) were purchased from Biosolve (Valkenswaard, The Netherlands). HPLC grade water was produced using a Milli-Q purification system from Millipore (Amsterdam, The Netherlands).

### 2.2. Instrumentation

#### 2.2.1. Microfluidic Confocal Fluorescence Detection System

The screening platform consisted of a nano-LC unit with a post-column split to an MS and a microfluidic chip acting as biochemical reactor, where the LC flow and a bioassay solution were mixed and incubated, and a microfluidic LED based laser-induced fluorescence (LIF) detector as described in detail in Heus *et al.* [39].

#### 2.2.2. Nano-LC and Solvent Delivery System

The Ultimate nano-LC system with a Famos autosampler was from LC Packings (Amsterdam, The Netherlands). The gradient system was operated at 400 nL/min. Mobile phase solvent A consisted of water/ACN 95:5 and 0.05% FA and solvent B of water/ACN 5:95 and 0.05% FA for *Conus textile* venom analysis. The toad extracts were analyzed with solvent A consisting of water and 0.1% TFA and solvent B of ACN and 0.1% TFA. Sample volumes of <500 nL were injected into the analytical capillary column (150 mm × 75 µm internal diameter (i.d.)) packed in-house with Aqua C18 particles (particle size 3 µm, 200 Å pore diameter; Phenomenex, Torrance, CA, USA). For *Conus textile* venom analysis, a 70 min gradient elution was applied running five min isocratic at 5% solvent B, then rising to 60% solvent B in 65 minutes. For *Bufo alvarius* and *Bufo marinus* analysis, a 70 min gradient elution was applied running five min isocratic at 1% solvent B, then rising to 60% solvent B in 65 min.

#### 2.2.3. Microfluidic Chip

The microfluidic chip and chip holder (type 4515), produced by Micronit Microfluidics, (Enschede, The Netherlands), was described in detail elsewhere [38]. One inlet was used to connect the nano-LC carrier flow; the other inlet to a Model 980532 syringe pump (Harvard Apparatus, Holliston, MA, USA) to infuse the AChBP and tracer ligand DAHBA at a flow rate of 5 µL/min, as described previously [37,38].

#### 2.2.4. Microfluidic LED Based LIF Detector

The flow cell of the detector consisted of a 150 µm i.d. extended light path “bubble cell” with 50 µm i.d. connecting capillaries (G1600 64232, Agilent Technologies, Amstelveen, The Netherlands). This bubble cell served as the actual flow-through detector cell. Light from a high-intensity LED passed a 465 nm single band pass filter, was collimated by a lens, reflected by a dichroic mirror under 90° and focused into the centre of the bubble cell by a 20× quartz microscope objective. Emitted light passed the same dichroic mirror, a focusing lens, and a 520 nm single band pass filter, after which it was detected by photomultiplier tube. A detailed description of this detector can be found in [38].

#### 2.2.5. Nano-LC-MS

A Shimadzu (‘s-Hertogenbosch, The Netherlands) ion-trap-time-of-flight (IT-TOF) hybrid mass spectrometer equipped with a Picoview nano-electrospray ionization (ESI) source from New Objective (Woburn, MA, USA) was operated in positive-ion mode. A 40 mm × 180 µm outer diameter × 30 µm i.d. stainless-steel emitter was used as the spray needle (ES522, Proxeon/Thermo Scientific, Waltham, MA, USA). The spray needle was connected to the nano-LC system via a 1,000 mm × 10 µm i.d. bare fused-silica capillary by a low void volume connector (type P 720, Upchurch Scientific, Oak Harbor, WA, USA) which was integrated in the nano-ESI source. The temperature of the heating block and curved desolvation line were set to 200 °C. The interface voltage was set at 1.7 kV, resulting in a current of ~32 µA.

#### 2.2.6. MS-Guided Fractionation

For purification, normal bore LC was performed using a 100 mm × 4.6 mm Symmetry Shield column at a flow rate of 0.5 mL/min. A Gilson 234 was used for sample injection (50 µL). The mobile phases used were the same as in nano-LC with the following gradient: 25 min at 2% B; 25 min linear increase to 95% B; five min at 95% B. By means of a post-column Y-split, 90% of the eluent was directed through a Shimadzu SPD-20A UV detector (220 nm, Shimadzu Crop., Kyoto, Japan) to a Gilson 234 fraction collector. Collected fractions were freeze-dried utilizing a centrifugal evaporator at room temperature. To the remaining 10% of the eluent, an additional flow of 50%/50% H_2_O/MeOH with 0.1% FA at 250 µL/min was mixed via a Shimadzu LC-10a pump to obtain favorable ESI flow rates and solvent composition. MS detection was done using ESI in the positive mode with a Q-TOF II mass spectrometer (Micromass, Manchester, UK). A 398 K source temperature, 573 K desolvation temperature, 250 L/h desolvation gas flow, 60 L/h cone gas flow, 17 psi gas cell pressure (*i.e.*, 20 V collision voltage for optimum transfer through collision cell), 3,500 V capillary voltage, and a cone voltage of 30 V were used. The mass range was *m/z* 50 to 2,000. The data acquisition parameters were 1.0 s spectrum^−1^ in MS profile mode and 0.1 s delay time. Nitrogen (purity 5.0; Praxair, Oevel, Belgium) and argon (purity 5.0; Praxair, Oevel, Belgium) were used as desolvation/cone gas and collision gas, respectively.

#### 2.2.7. Off-Line NMR

NMR spectra were recorded on a Bruker Avance III spectrometer equipped with a five mm Cryo Probe CPTXI with z-axis gradients and automatic tuning and matching accessory (Bruker Biospin, Rheinstetten, Germany). The spectrometer was operated via Bruker TopSpin 2.1 software, running under CentOS. Data was processed using TopSpin 2.1 running under Linux. The resonance frequency for ^1^H NMR was 599.76 MHz and for ^13^C 150.82 MHz. All spectra were recorded in methanol-d_4_ at 298 K. Standard Bruker parameter settings—with the pulse sequences indicated in brackets according to the Bruker nomenclature—were used for recording of ^1^H (zgpr), ^1^H-^1^H COSY (cosygpprqf), ^1^H-^1^H TOCSY (mlevphpr), ^1^H-^13^C HSQC (hsqcetgpsi) and ^1^H-^13^C HMBC (hmbcpgndqf) NMR spectra. Presaturation of the residual water resonance was performed prior to the ^1^H-1D and ^1^H-2D NMR experiments. All 2D NMR experiments were recorded with 2K data points in the direct dimension and 128 data points in the indirect dimension. The spectra were processed using zero filling such that data matrices of 2 K × 1 K were obtained. The spectra were calibrated against the residual solvent signal of methanol-d4; δ ^1^H 3.31 ppm, δ ^13^C 49.0 ppm.

### 2.3. Biochemical Assays and Samples

#### 2.3.1. Biochemical Assay

Fresh solutions of 5 nM Ls-AChBP and 15 nM DAHBA were made every day by dissolving in a bioassay solution containing 1 mM KH_2_PO_4_, 3 mM Na_2_HPO_4_, 0.16 mM NaCl, 20 mM trizma base/HCl at Ph 7.5 and 400 µg/mL ELISA BR. The bioassay solution was kept in a 2.5 mL syringe (type 1002LTN, Hamilton, Bonaduz, Switzerland) at 4 °C. The on-line bioassay itself was performed at 22 °C [38].

#### 2.3.2. Radioligand Binding Assay

RBAs for human α7-nAChR were performed as reported previously [34,40]. α7-Receptor expressing SH-SY5Y cells cells were harvested, washed with PBS and washed three times with ice-cold PBS by centrifugation at 4 °C. Aliquoted cell pellets were stored at −80 °C. Frozen cell pellets were dissolved in ice-cold PBS, homogenized and sonicated immediately before use and added to the assay. The final volume of binding buffer suspension with nAChR-rich membranes from SH-SY5Y cells was 100 µL. The buffer was a 1.4 mM KH_2_PO_4_, 4.3 mM Na_2_HPO_4_, 137 mM NaCl, 2.7 mM KCl, 20 mM Trizma base, 0.05% Tween 20 buffer at pH 7.4. As radioligand, ^3^H-MLA (K_D_ = 1.81 nM) was used at a concentration of 2.5 nM. The SH-SY5Y homogenate was incubated with 10^−3^–10^−1^ M of ligand. Crude *Bufo alvarius* skin extracts were dissolved at 300 µg of crude mixture per 100 µL assay buffer, for an (arbitrary) compound concentration of 10^−3^ M, assuming skin extract compounds average at 300 Dalton. After an incubation period of 1.5 h under continuous shaking, bound radioligand was collected on 0.3% polyethyleneimine-pretreated Unifilter-96 GF/C filters (Perkin Elmer, Waltham, USA) and washed using ice-cold washing buffer (4 °C) consisting of 50 mM Tris-HCl buffer at pH 7.5. After the filters were dried, scintillation fluid (MicroScint, Perkin Elmer) was added after which radioactivity was measured in a Wallac 1450 MicroBeta liquid scintillation counter (Perkin Elmer). To determine non-specific binding, radioligand saturation experiments were performed with nicotine. The concentration of nicotine used was 1 mM. Binding assay data was analyzed using Prism 5.0 (Graphpad Software, Inc., San Diego, California, USA).

#### 2.3.3. *Conus textile* Venom

Lyophilized venom sample from *Conus textile* was acquired as described by Dutertre *et al.* [41]. 1 mg of lyophilized venom was dissolved in water/MeOH 95:5% at a concentration of 5 mg/mL and subsequently centrifuged at 13,400 rpm for 10 min to remove particulate matter. Aliquots of these samples were stored at −20 °C until use. Before analysis, nicotine was added as internal standard at an end concentration of 40 µM.

Venom peptide cysteine bridges were reduced by adding 2 µL of the 5 mg/mL venom mixture to 18 µL reduction solution, which was then allowed to react at 50 °C for 45 min. The reduction solution contained 1 M DTT and 2 M guanidine-HCl, buffered at pH 8.5 by 50 mM NH_4_HCO_3_. The reduced venom was then analyzed by nano-LC-MS. During the LC-MS analysis of the reduced venom mixture, the nano-LC unit remained hyphenated to the bioaffinity screening setup. This served as an indication whether the reduced peptide(s) remained bioactive. Full MS spectra were acquired between *m/z* 150 and 3,000. Data-dependent MS^2^ data were obtained with a precursor ion isolation width of 3.0 atomic mass units and product ions between *m/z* 150 and 2,000. In a subsequent analytical run, MS^2^ data was targeted for the reduced α-TxIA peptide at *m/z* 831.360 (from ions obtained between *m/z* 150 and 1,800 in the ion trap). MS^2^ product-ion *m/z* values were compared with theoretical α-TxIA peptide fragments as calculated by the University of California, San Francisco’s (UCSF’s) protein prospector. Theoretical fragment calculation was based on the α-TxIA sequence as published by Dutertre *et al.* [41].

#### 2.3.4. Toad Skin Excretion Extracts

Lyophilized skin secretion samples from *Bufo alvarius* and *Bufo marinus* were dissolved in water/ACN 99:1 and 0.1% TFA at a concentration of 10 mg/mL and subsequently centrifuged at 13,400 rpm for 10 min. Aliquots of these samples were stored at −20 °C until further use. Before analysis, three reference peptides at an end concentration of 2 µM each were added. The resulting solutions were directly injected in duplicate onto the nano-LC system for parallel bioaffinity screening and MS identification. Samples were re-analyzed at lower or higher concentrations whenever necessary. The three reference peptides were used to align MS data between different runs, whenever necessary. The three reference peptides were [Met^5^] enkephalin, human angiotensin I, and Mca-Lys-Pro-Leu-Gly-Leu-Dap(Dnp)-Ala-Arg-NH_2_, which were detected at *m/z* 627.146, 648.848, and 611.307, respectively.

## 3. Results and Discussion

This work describes the application of an efficient analytical HRS platform for the bioaffinity profiling and subsequent MS-guided purification of compounds from natural sources. Previously, we showed its applicability to the bioaffinity profiling of neurotoxic snake peptides (between 60 and 90 amino acids) for Ls-AChBP binding [39]. Here, profiling is demonstrated for small peptides and small bioactive molecules in neurotoxic cone snail venoms and in extracts of toad skin excretions, respectively. In this extended workflow, the toxins identified with the miniaturized HRS platform were subsequently isolated by “normal-bore” LC using MS-guided fractionation. Thereafter, isolated small molecular ligands were structurally elucidated by NMR and biologically assessed using an RBA for assessing human α7-nAChR binding.

**Figure 1:**
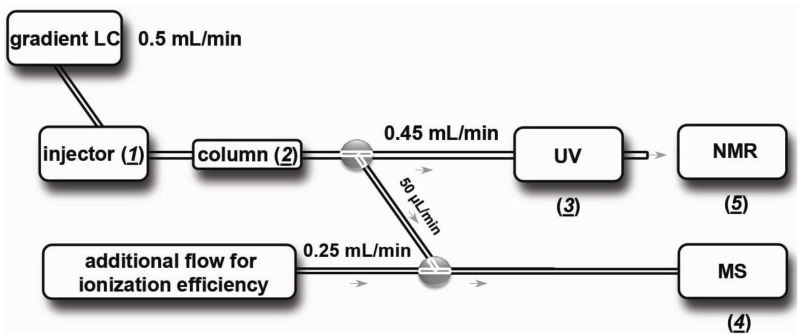
Schematic diagram of the MS-guided purification. Venom is injected for analysis (***1***). After (large bore) LC (***2***), a split allows eluting toxin constituents to collection vials for NMR analysis (***3***) with help of a 1:9 post-LC split and to go to ESI-MS for monitoring of toxin mass (***4***).

The miniaturized analytical screening setup was used as described by Heus *et al.* [39]. Half of the 400 nL/min effluent from the nano-LC is split to the nano- ESI interface. The other half is hyphenated to the microfluidic chip where it is continuously mixed with the bioassay solution, which is infused at 5 µL/min by a syringe pump. The chip outlet is connected to the confocal LED-induced fluorescence detector, which monitors the eluting toxins for bioaffinity after in-flow and in-chip incubation. The biochemical assay is based on fluorescence enhancement in which the tracer ligand DAHBA shows increased fluorescence when bound to the AChBP. Eluting ligands, when competing with DABHA for Ls-AChBP binding, are observed as a negative peak. Identified bioactives may then be subsequently purified by normal-bore LC-MS, guided by the accurate mass of the ligands, for further pharmacological studies on human nAChRs. This subsequent MS-guided purification setup is shown in Figure 1.

### 3.1. Method Evaluation for Cone Snail Venoms

**Figure 2:**
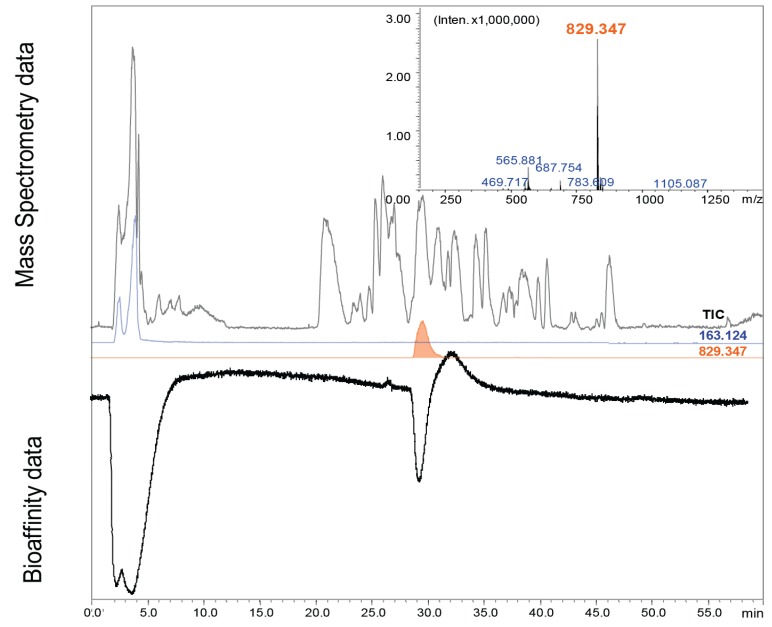
Typical result of a crude snail venom analysis where 2.5 μg of crude *Conus textile* venom was injected in a 500 nL sample. High resolution MS^1^ analysis and on-line bioassay data were obtained in parallel. This analytical run obtained one ligand, identified as α-TxIA by the high resolution MS^1^ analysis. 40 µM nicotine functioned as an alignment compound between the MS and the bioassay trace (the split nicotine peak is due to the addition of 5% MeOH to the sample; the chromatographic run started at 5% ACN). The binding signal in the bioassay trace aligned with the doubly charged ion with *m/z* 829.347 (mass spectrum is shown in the insert), which corresponds to a monoisotopic mass of 1,656.678 Da.

To evaluate the applicability and performance of the miniaturized methodology for the screening of bioactive peptides in snail venom, a dilution series of the venom was first analyzed in order to assess sensitivity, repeatability, and correlation of peak shapes observed in MS and the biochemical assay, as well as the (post-column) band broadening. This was done by injecting 0.1, 0.5 and 2.5 μg of *Conus textile* venom in a 500 nL sample (Supporting Information, Figure S1). In *Conus textile* venom, one AChBP ligand was found. The analysis of 2.5 μg venom showed some peak broadening, but not as extreme as was observed for some high-affinity snake toxins [39]. Previously, we demonstrated that a purified cone snail (*Conus imperialis*) peptide was detected with high sensitivity and with not much peak broadening in the bioassay. Some tailing in the bioassay is observed, which is common for on-line bioassays as the response is sigmoidal (see Heus *et al.* [39]). Here, we found a similar peak shape in the bioassay of the crude *Conus textile* venom analysis for the bioactive peptide. From parallel MS analysis, the correlated *m/z* of the bioactive peptide was rapidly identified, as shown in Figure 2. The peak in the extracted ion chromatogram (EIC) of the ion with *m/z* 829.347 correlated to the bioassay peak of the cone snail peptide. The estimated limit of detection of the bioactive peptide was around 0.5 μg venom in 0.5 μL injected.

### 3.2. Cone Snail Toxin Screening

**Figure 3:**
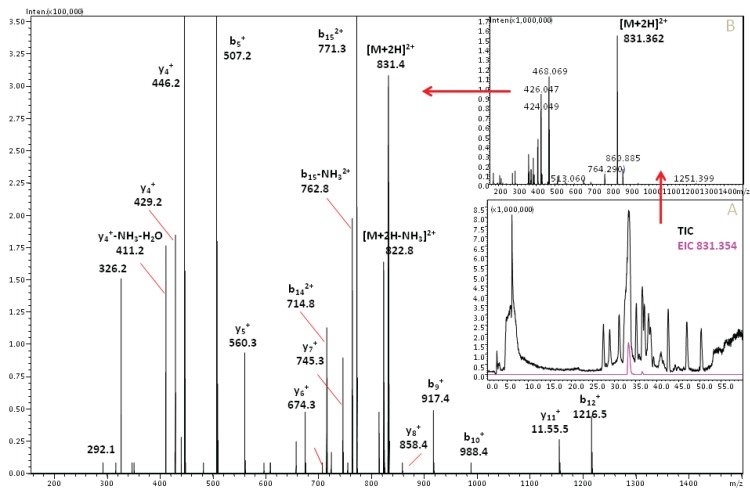
Data-dependent MS^2^ spectrum analysis of the reduced α-TxIA peptide. Fragments that can be attributed to α-TxIA are marked. The doubly charged peptide with *m/z* 831.360 results in a peptide mass of 1,661.689 Da, which is in good agreement with the previously published mass of 1,661.67 Da of α-TxIA. The inserts show the total ion current chromatogram and the extracted ion current of *m/z* 831.354 (insert A) and the MS^1^ spectrum of the corresponding chromatographic fraction (insert B).

Correlation of the MS data with the bioaffinity data (Figure 2) conveniently identified the binding conotoxin peptide amongst the over 1,000 peptides present in the cone snail venom [2]. The 2^+^-ion with *m/z* 829.347 (Figure 2; insert) matches with the known α7-nAChr ligand α-TxIA with (GCCSRPPCIANNPDLC, amidated *C*-terminal, theoretical *m/z* of [M+2H]^2+^ is 829.338, mass error +9 mDa), found by Dutertre *et al.* [41]. For further confirmation of the peptide identity, the whole venom was reduced with DTT and then analyzed in data-dependent MS^2^ analysis (Figure 3). The ion with *m/z* 829.347 was no longer found, but a 2^+^-ion with *m/z* 831.359 (mass error +6 mDa) emerged, *i.e.*, the original peptide *m/z* + 2, which corresponds to an accurate peptide mass of 1,661.694 Da. Figure 3 shows the MS^2^ spectrum of this peptide in the venom extract and in the inserts the total ion current (TIC) chromatogram (A) and the MS^1^ spectrum of the relevant chromatographic peak (B). The *m/z* + 2 after reduction in the 2^+^-ion indicates the reduction of two cysteine bridges, which is in line with the peptide α-TxIA. The partial sequence of the peptide can be derived from the MS^2^ spectrum, as shown in Figure 3. In fact, this experiment shows a strategy for confirmation of the presence of disulfide bridges of a found bioactive peptide, based on reduction of all disulfide bridges: after reduction by DTT, the bioactive peak should disappear, and another peak should appear somewhere in the chromatogram, featuring an *m/z* shift corresponding to the number of disulfide bridges present. Sequencing of this reduced peptide can be achieved by MS^2^. As we confidently identified the peptide as being the α-TxIA peptide based on accurate mass, the presence of the two expected disulfide bridges and the (partial) sequence information from the MS^2^ data (see Figure 3), and because the peptide was already characterized by Dutertre *et al.* [41], we decided not to proceed with subsequent purification in this case.

### 3.3. Screening of Toad Skin Excretions

**Figure 4:**
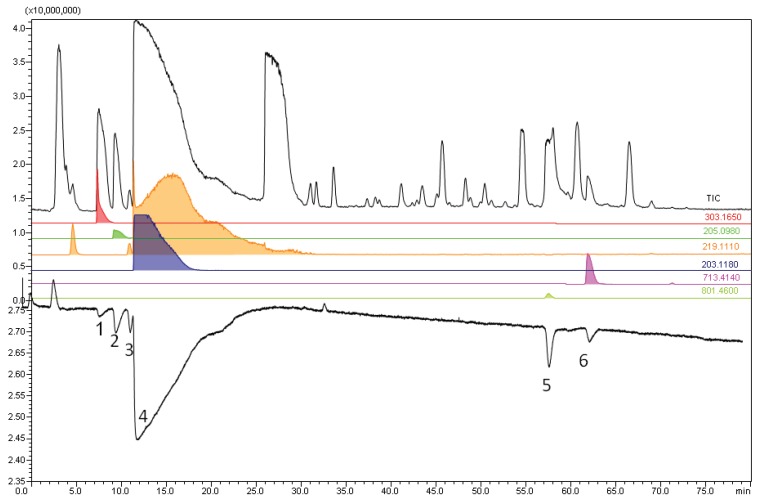
An analysis of a 500 nL sample containing 5 µg *Bufo marinus* lyophilized skin secretion extract obtained six binding signals.

For the screening of toad skin excretion extracts, no nicotine was added to the samples as it could interfere with the detection of small-molecule ligands. In the lyophilized skin excretions (5 μg) of the *Bufo marinus* and *Bufo alvarius*, a total of six AChBP ligands were found. Both species exhibited almost identical binding profiles, as shown in Figure 4 and Figure S2 in the supporting information for *Bufo marinus* and *Bufo alvarius*, respectively. The ligands found in *Bufo marinus* were fractionated by normal-bore LC-MS. The advantage of using MS-guided purification is that with MS analysis we can plot EICs of *m/z* values seen in the area of fraction collection. Plotting the EICs can show the peak purity of our collected fractions, while UV detection does not give information about the purity of the eluting peaks. Once peak purity is known, one can subsequently revert to UV based fractionation as the data obtained from LC-UV-MS can be translated to LC-UV for further easy, cost effective and convenient LC-UV based purification. The ligands found in *Bufo marinus* were fractionated by normal-bore LC. For the fraction collection 50 µL 10 mg/mL Bufo marinus skin extract sample was injected to the LC. The isolated compounds were then analyzed in a RBA to assess real α7-nAChR binding. Again, AChBP proved to be a valid homologue of the α7-nAChR binding pocket, as the identified ligands showed binding affinity to the α7-nAChR in radioligand displacement assay. NMR data were acquired for additional structural elucidation/confirmation.

In MS analysis, the first bioactive compound (at a retention time of 8.5 min) showed an ion with *m/z* 303.168, consistent with a most-likely formula of C_12_H_23_N_4_O_5_^+^ (mass error −1.9 mDa). Although an information-rich MS^2^ spectrum was obtained, we did not succeed in interpreting the data to retrieve the identity of this compound (Supporting Information, Figure S3). Structure proposals of the second (minor) bioactive (10.0 min; *m/z* 205.098; C_11_H_13_N_2_O_2_^+^; +0.8 mDa) and third bioactive compound (10.5 min; *m/z* 219.112; C_12_H_15_N_2_O_2_^+^; −0.7 mDa) could only be made upon interpretation of the MS^2^ spectra (Figures S4 and S5 in supporting information). Both compounds, assuming the structure proposals are correct, have not been described in relation to this toad venom. Unfortunately, for these three minor bioactivity peaks, too many co-eluting compounds were present to obtain sufficiently pure fractions for unambiguous NMR analysis.

**Figure 5:**
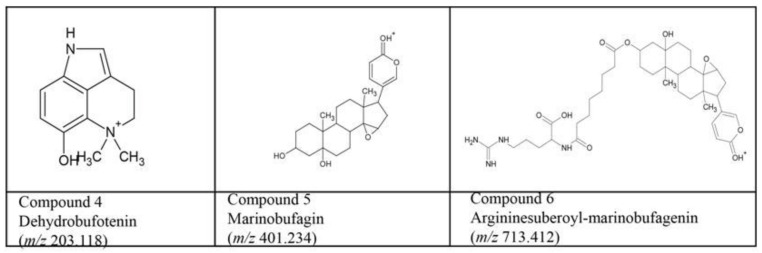
Structures of the identified bioactives from *Bufo marinus* skin secretion extract, confirmed by both MS^n^ and NMR.

The fourth bioactive compound (*m/z* 203.118; C_12_H_15_N_2_O^+^; mass error within 1 mDa), co-eluting with compound **3**, was identified as dehydrobufotenin (Figure 5), a well-documented bufotoxin [42]. In MS^2^, the subsequent losses of two methyl radicals (CH_3_^●^) confirmed the dimethyl-substituted quaternary ammonium character of this compound (Supporting Information, Figure S6). NMR analysis confirmed these findings (see Section 3.4 and Supporting Information, Figure S7A–C).

The fifth bioaffinity signal at 58.0 min correlated to a compound that was identified as marinobufagin (*m/z* 401.234; C_24_H_33_O_5_^+^, mass error +5 mDa; in some cases appearing as a proton-bound dimer [2M+H]^+^ with *m/z* 801.460), also reported by Gao *et al.* [43] (Figure 5). The MS^2^ spectrum (Supporting Information, Figure S8) is in agreement with data reported earlier [14,44]. NMR data supported these findings (see Section 3.4. and Supporting Information, Figure S9A,B).

The sixth bioaffinity signal at 62.0 min correlated to a compound with *m/z* 713.414 (C_38_H_57_N_4_O_9_^+^, mass error +2 mDa). The molecular formula is consistent with arginine-suberoyl marinobufagenin, reported by Yoshika *et al.* [45] (Figure 5). The MS^2^ spectrum shows the expected loss of the marinobufagenin part of the molecule with charge retention on the arginine-suberoyl part (Supporting Information, Figure S10), consistent with data reported by Yoshika *et al.* [45]. Subsequent losses involve typical small molecule losses (H_2_O, CO, NH_3_, and CH_2_N_2_) expected for the arginine-suberoyl part. A definitive confirmation of the structure by NMR was not possible because of the low signal-to-noise ratio of the NMR spectrum (see Section 3.4).

### 3.4. NMR Data on Toad Fractions

Compounds analyzed by NMR are numbered below according to the description in the results and discussion based on the nano-LC elution order.

*Compound* **4**: (*m/z* 203.118) The proton spectrum of compound **4** exhibited three characteristic dehydrobufotenin signals in the aromatic region at δ 7.33 ppm (1H, d, *J* = 8.6 Hz), δ 7.15 ppm (1H, s) and δ 6.83 ppm (1H, d, *J* = 8.6 Hz). In the aliphatic region, two methylene signals at δ 4.06 ppm (2H, t, 5.9 Hz) and δ 3.33 ppm (2H, t, 5.9 Hz) and two methyl signals both at δ 3.73 ppm (6H, s) were observed, confirming the structure to be dehydrobufotenin. Carbon assignments were corroborated by ^1^H,^13^C-HSQC and ^1^H,^13^C-HMBC experiments (Supporting Information, Figure S7A–C).

*Compound* **5**: (*m/z* 801.460; proton-bound dimer) compound **5** has been assigned as marinobufogenin. The essential signals in ^1^H-1D and ^1^H-2D spectra are the α-pyrone signals at δ 7.90 ppm (1H, dd, *J* = 9.7, 2.4 Hz), δ 7.45 ppm (1H, dd, *J* = 2.4, 1.0 Hz) and δ 6.26 ppm (1H, dd, *J* = 9.7, 1.0 Hz), the epoxy signal 15 CH at δ ^1^H 3.61 ppm (1H, d, *J* = 8.5 Hz) and two methyl signals at δ 0.79 ppm (3H, s) ppm and δ 0.97 ppm (3H, s). The specific carbon resonances were observed in ^1^H,^13^C-HSQC or ^1^H,^13^C-HMBC at δ 60.7, 75.7 ppm for the epoxy and δ 124.3, 164.6 ppm for the α-pyrone. Almost all of the steroidal signals are doubled as also seen in compound **6**. Compound **5** and **6** were most probably purified as two co-eluting fractions as compound **6** was found in compound **5**, and vice versa. As this did not interfere with the NMR elucidation and NMR was performed to assist the MS measurements, further purification attempts were not done. Although the resonance of 18CH_3_ were also doubled, most of the extra signals originated from a modification around C3/C5 as could be observed in 2D TOCSY, HSQC and HMBC data (Supporting Information, Figure S9A,B).

*Compound* **6**: (*m/z* 713.414) Only the steroidal specific shifts for 18CH_3_ and 19CH_3_ at resp. δ 0.79 ppm and δ 1.00 ppm were observed for compound **6**. Those chemical shifts are close to those of marinobufagenin (compound **5**) but slightly modified, probably in the A or B-ring. The signals of the low content compound **6** are doubled in a 60/40-ratio. The major signals are in the aromatic region equal to the signals of compound **5**. In addition, the ^1^H chemical shift at 0.78 ppm corresponding to 18CH_3_ is identical to the one in compound **5**. The signal of H15 was not observed at the same shift. The chemical shift of 19CH_3_ at 1.00 ppm is slightly higher than the observed 19CH_3_ shift in marinobufagenin at 0.97 ppm. This might indicate that the suberoyl is added at position 3. Nevertheless, the compound is marinobufagin-like and modified in the A and B-ring indicating *di*-suberoyl marinobufagin, which is in accordance with the MS data.

### 3.5. Bufo alvarius Bioaffinity Assessment

Our current HRS approach aims at rapid assessment of binders. Therefore, the (usually low) amount of each bioactive compound isolated can only be roughly estimated and a quantitative assessment of relative binding potencies cannot be made. We estimate that between 0.05 and 0.5 mg compound was collected, which is sufficient for NMR analysis and an initial assessment of binding to nAChR in a RBA. First, the crude *Bufo alvarius* skin excretion sample was analyzed by a traditional RBA for α7-nAChR to assess binding potency of included compounds. This showed 50% binding at a concentration of ~3 mg/mL. Serial dilutions were prepared for the purified fractions 1, 2, 4, 5 and 6. For all fractions, RBA show binding to nAChR, except for fraction 6 (at the current total amount of compound isolated and re-dissolved).

## 4. Conclusions

This work demonstrates that the developed miniaturized on-line HRS system was able to identify a bioactive peptide in the *Conus textile* with AChBP affinity amongst >1,000 (small) peptides. The current methodology was able to identify the bioactive conotoxin within two rapid analytical runs (60 min each). It does so robustly and is able to use minute amounts of venom (2.5 μg). Therefore, the HRS platform enables efficient screening for bioactives in natural extracts which are available only in low sample amounts, e.g., spider and scorpion venoms. To show that this platform is also applicable to screen non-peptide small compounds (molecular weight of 200 to 1,000 Da), skin secretions from the Colorado River toad (*Bufo alvarius*) and Cane toad (*Bufo marinus*) were analyzed. The results demonstrated several tryptamine-like and steroidal ligands, which, to our knowledge, have as of yet never been correlated with AChBP (and/or nAChR) affinity. By extending our workflow with a rapid analytical purification, NMR analysis, and rescreening in either the miniaturized screening system or a conventional RBA, we were able to isolate bioactive toxins from natural extracts in a straightforward and sample conserving way for initial and rapid chemical and biochemical assessment.

## Abbreviations

α7-nAChR: α7-nicotinic acetylcholine receptor
^3^H-MLA: ^3^H-methyllycaconitine
AChBP: Acetylcholine binding protein
DAHBA: (*E*)-3-(3-(4-diethylamino-2-hydroxybenzylidene)3,4,5,6-tetrahydropyridin-2-yl)pyridine
DTT: DL-dithiothreitol
ESI: Electrospray Ionization
EICs: Extracted Ion Chromatograms
FA: Formic Acid
HRS: High-Resolution Screening
i.d.: Internal diameter
IT TOF: Ion-Trap-Time-of-Flight
Ls: *Lymnaea stagnalis*
LC: Liquid Chromatography
MeOH: Methanol
RBA: Radioligand Binding Assay
TIC: Total Ion Chromatogram
TFA: Trifluoroacetic acid

## Supplementary Files

Supplementary File 1 Supplementary Materials (PDF, 971 KB)
