# Supplementary material for: Miniaturized Bioaffinity Assessment Coupled to Mass Spectrometry for Guided Purification of Bioactives from Toad and Cone Snail
Source: Biology (Basel). 2014 Feb 13;3(1):139–56. doi: 10.3390/biology3010139 (PMC4009767; doi:10.3390/biology3010139)
Supplement: Supplementary File 1 — Supplementary Materials (PDF, 971 KB) [file biology-03-00139-s001.pdf]

Supplementary Materials

Miniaturized Bioaffinity Assessment Coupled to Mass Spectrometry for Guided Purification of Bioactives from Toad and Cone Snail

Ferry Heus [1,†], Reka A. Otvos [1,2,†], Ruud L.E.G. Aspers [3], Rene van Elk [2], Jenny I. Halff [1], Andreas W. Ehlers [4], Sébastien Dutertre [5], Richard J. Lewis [5], Sybren Wijmenga [3], August B. Smit [2], Wilfried M.A. Niessen [1,6] and Jeroen Kool [1,*]

[1] AIMMS Division of BioMolecular Analysis, Faculty of Sciences, VU University Amsterdam, De Boelelaan 1081, 1083 HV Amsterdam, The Netherlands; E-Mails: ferryheus@gmail.com (F.H.); r.a.otvos@vu.nl (R.A.O.); j.i.halff@student.vu.nl (J.I.H.); w.m.a.niessen@vu.nl (W.M.A.N.)

[2] Department of Molecular and Cellular Neurobiology, Center for Neurogenomics and Cognitive Research, Neuroscience Campus Amsterdam, VU University Amsterdam, De Boelelaan 1085, 1081 HV Amsterdam, The Netherlands; E-Mails: rene.van.elk@vu.nl (R.E.); guus.smit@vu.nl (A.B.S.)

[3] Department of Biophysical Chemistry, Institute for Molecules and Materials, Radboud University Nijmegen, Heyendaalseweg 135, 6525 AJ Nijmegen, The Netherlands; E-Mails: r.aspers@nmr.ru.nl (R.L.E.G.A.); s.wijmenga@nmr.ru.nl (S.W.)

[4] AIMMS Division of Organic Chemistry, Faculty of Sciences, VU University Amsterdam, De Boelelaan 1081, 1083 HV Amsterdam, The Netherlands; E-Mail: a.w.ehlers@vu.nl

[5] The Institute for Molecular Bioscience, The University of Queensland, St Lucia, Queensland 4072, Australia; E-Mails: s.dutertre@imb.uq.edu.au (S.D.); r.lewis@imb.uq.edu.au (R.J.L.)

[6] hyphen MassSpec Consultancy, de Wetstraat 8, 2332 XT Leiden, The Netherlands

[†] These authors contributed equally to this work.

[*] Author to whom correspondence should be addressed; E-Mail: j.kool@vu.nl; Tel.: +31-205987542; Fax: +31-205987543

Received: 9 December 2013; in revised form: 23 January 2014 / Accepted: 26 January 2014 / Published: 13 February 2014

Figure S1. On-line bioassay analysis of three consecutive 500 nL sample injections containing 0.1, 0.5 and 2.5 µg crude *Conus textile* venom. The 0.1 and 0.5 µg venom injections also contained 40 µM nicotine to align the MS and bioassay trace.

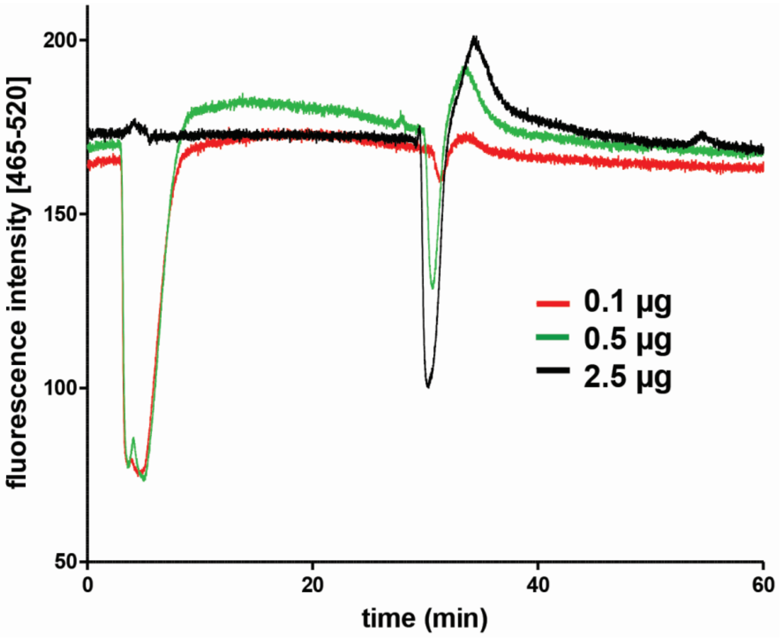

Figure S2. An analysis of a 500 nL sample containing 5 µg *Bufo alvarius* skin secretion extract obtained identical binding signals and attributed masses as the *Bufo marinus* extract analysis.

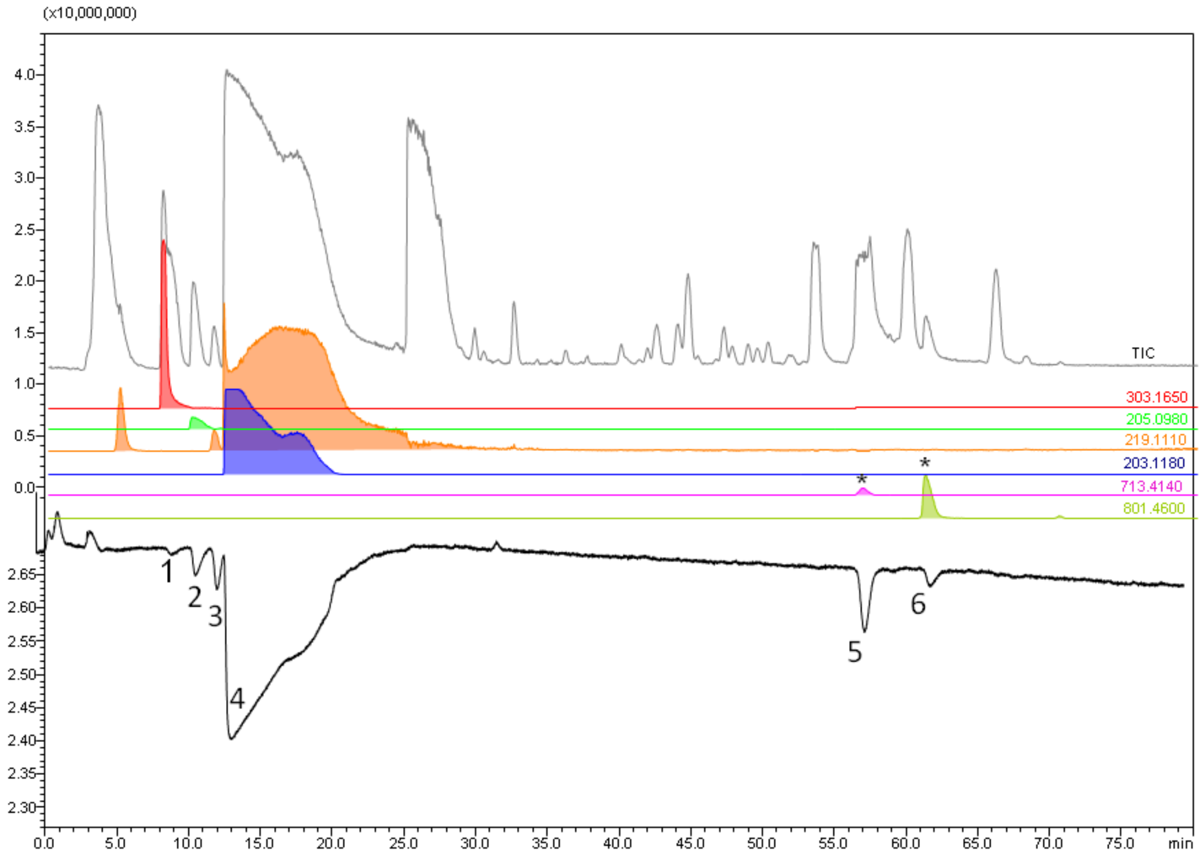

Figure S3. MS^2 spectrum of the unknown bioactive compound with *m/z* 303.168, eluting at 8.5 minutes. In the table below Figure S3, the proposed molecular formula, and proposed fragmentation of the compound is shown.

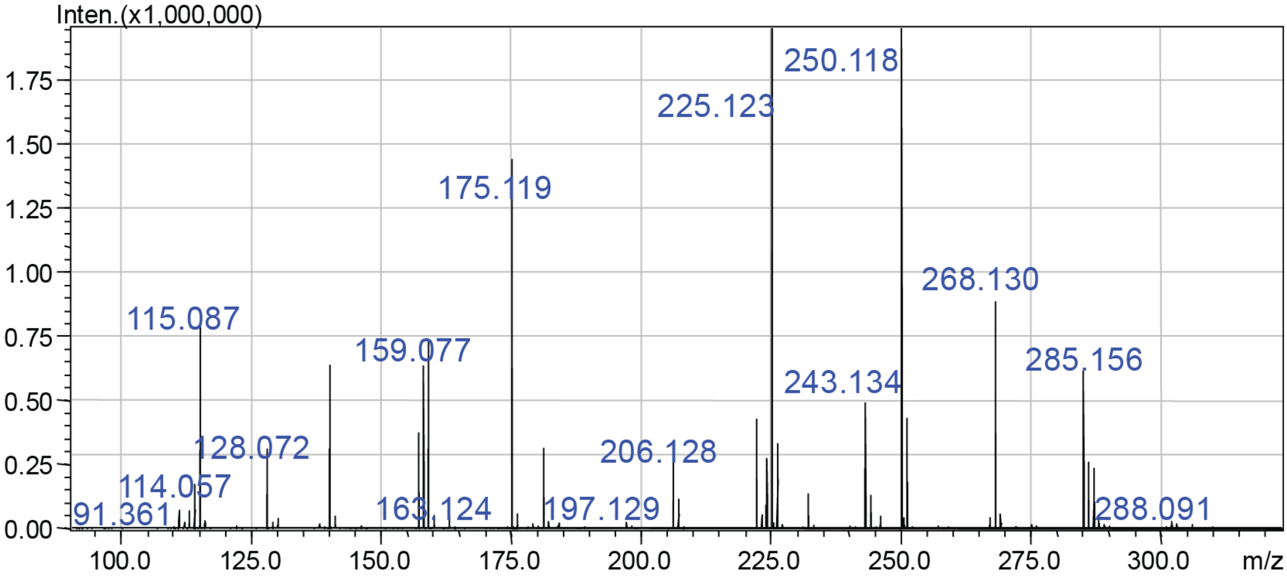

m/z	Formula	Interpretation
303.168	$C_{12}H_{23}N_4O_5^+$	$[M+H]^+$
285.156	$C_{12}H_{21}N_4O_4$	Loss of H_2O
**268.130**	$C_{12}H_{18}N_3O_4$	Loss of H_2O and NH_3
**250.118**	$C_{12}H_{16}N_3O_3$	Loss of 2 ×H_2O and NH_3
243.134	$C_{11}H_{19}N_2O_4$	Loss of CH_4N_2O, e.g., NH_3 and HNCO
**225.123**	$C_{11}H_{17}N_2O_3$	Loss of H_2O from *m/z* 243
181.133	$C_{10}H_{17}N_2O$	Loss of $C_2H_6N_2O_4$, e.g., 2xH_2O and 2xHNCO
**175.119**	$C_6H_{15}N_4O_2$	Loss of $C_6H_8O_3$
159.077	$C_6H_{11}N_2O_3$	Loss of $C_6H_{12}N_2O_2$
140.082	$C_6H_{10}N_3O$	Loss of $C_6H_{13}NO_4$
128.072	$C_6H_{10}NO_2$	Loss of $C_6H_{13}N_3O_3$
115.087	$C_5H_{11}N_2O$	Loss of $C_7H_{12}N_2O_4$ or $C_6H_{12}N_2O_2$ and CO_2
91.361		Not consistent, too big mass defect

Figure S4. (**A**) MS^2 spectra of the bioactive compound with *m/z* 205.098, eluting at 10.0 min. (**B**) The proposed structure, and proposed fragmentation scheme of the bioactive compound with *m/z* 205.098, eluting at 10.0 min.

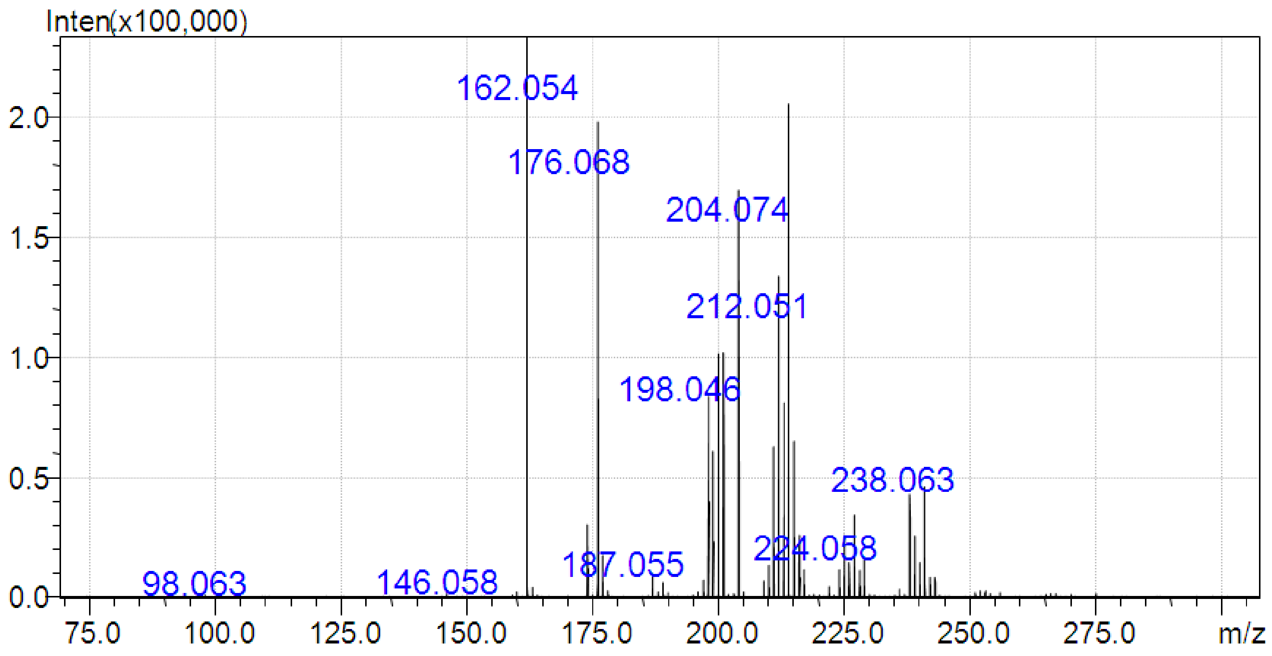

(**A**)

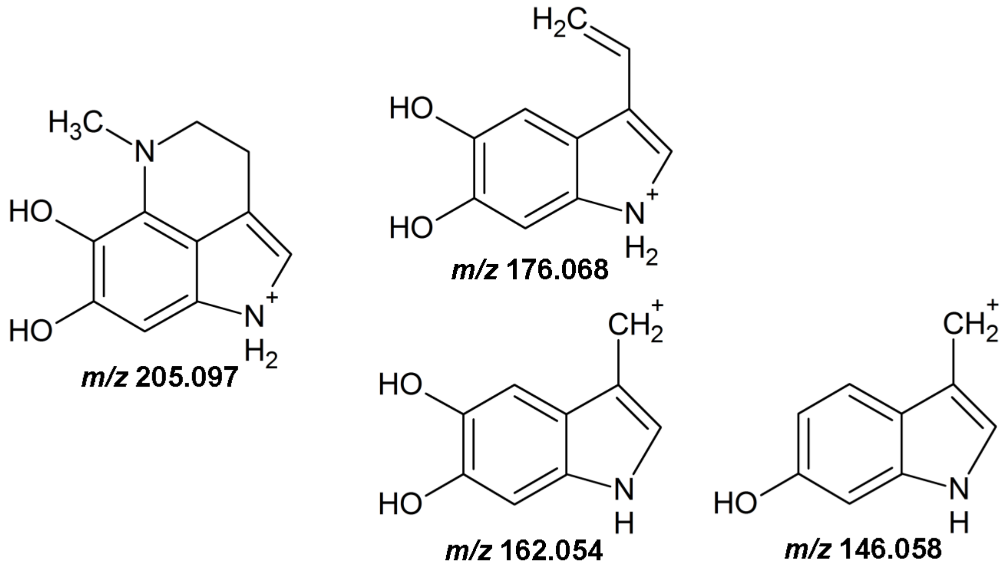

(**B**)

Figure S5. (**A**) MS^2 spectrum, proposed structure, and proposed fragmentation scheme of the bioactive compound with *m/z* 219.112, eluting at 10.5 min. (**B**) Proposed structure and proposed fragmentation scheme of the bioactive compound with *m/z* 219.112, eluting at 10.5 min.

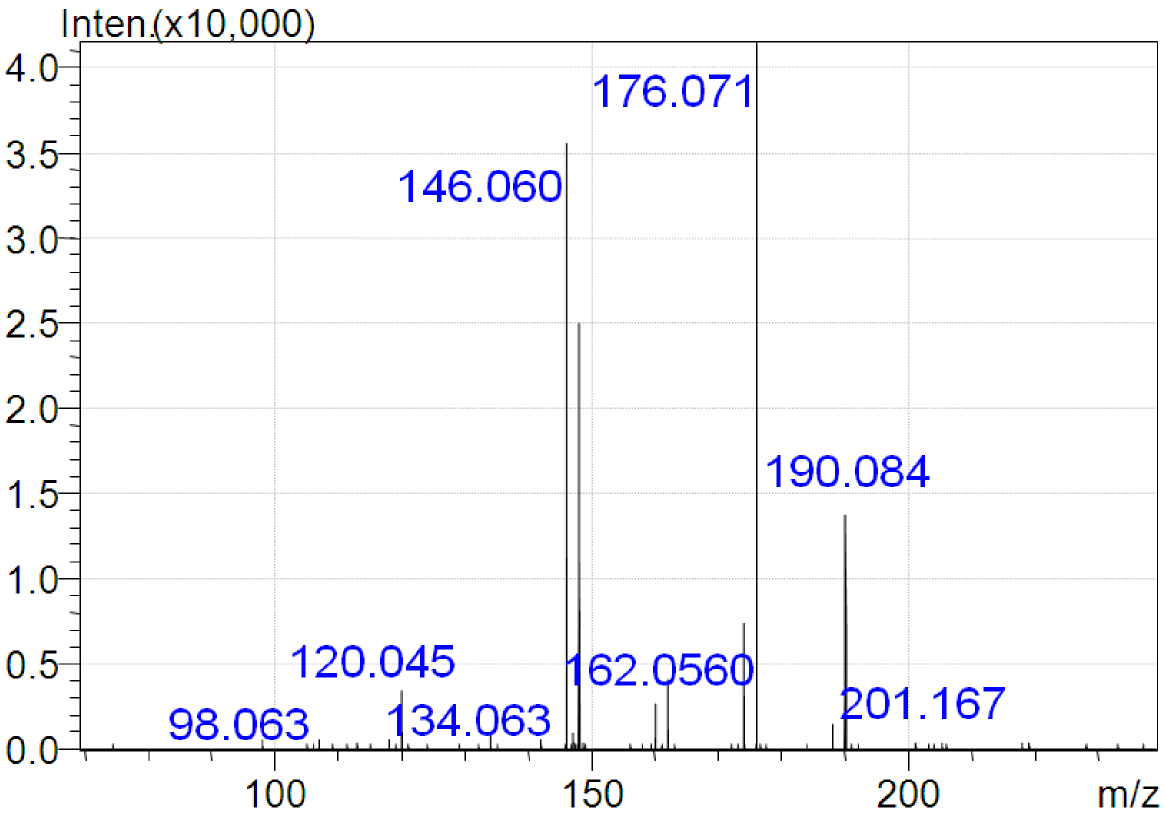

(**A**)

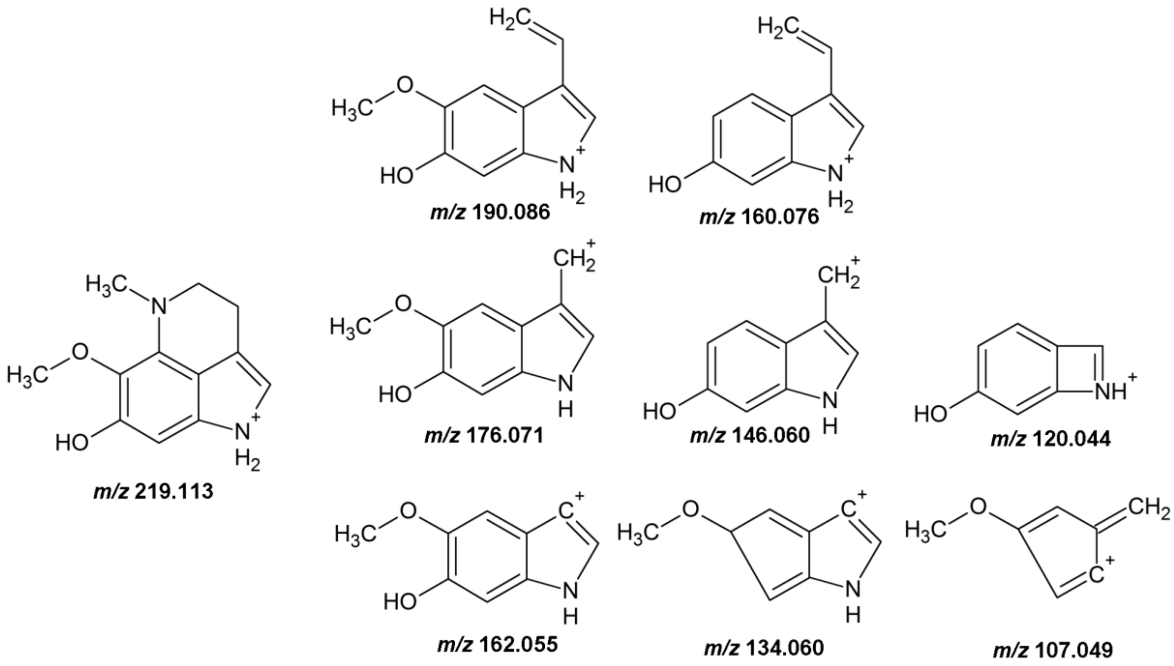

(**B**)

Figure S6. (**A**) MS^2 spectrum, proposed structure, and proposed fragmentation scheme of the bioactive compound with *m/z* 203.118, eluting at 10.5 min. (**B**) Proposed structure, and proposed fragmentation scheme of the bioactive compound with *m/z* 203.118, eluting at 10.5 min.

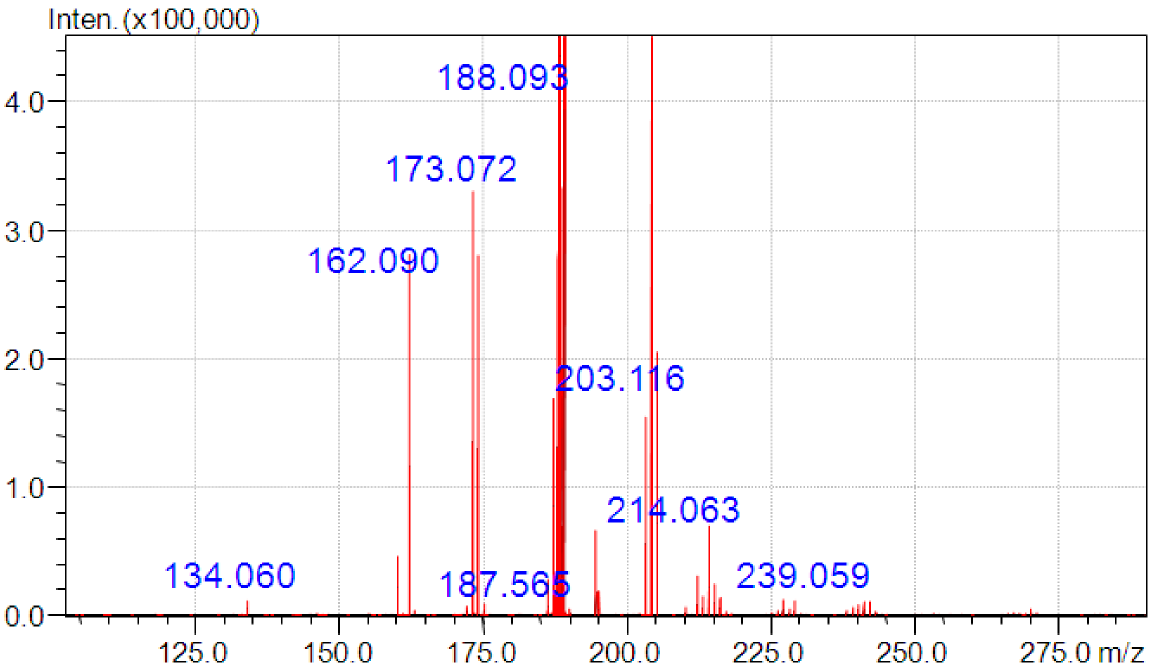

(**A**)

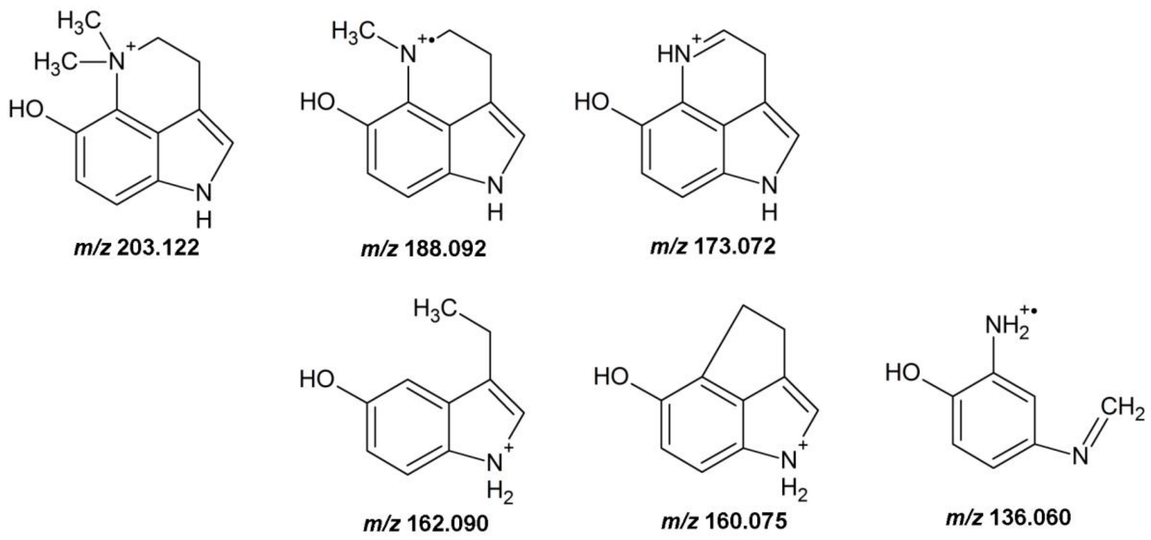

(**B**)

Figure S7. (**A**) HMBC-NMR spectrum of the bioactive compound with *m/z* 203.118, eluting at 10.5 min. (**B**) HSQC-NMR spectrum of the bioactive compound with *m/z* 203.118, eluting at 10.5 min. (**C**) HSQCHMBC-NMR spectrum of the bioactive compound with *m/z* 203.118, eluting at 10.5 min.

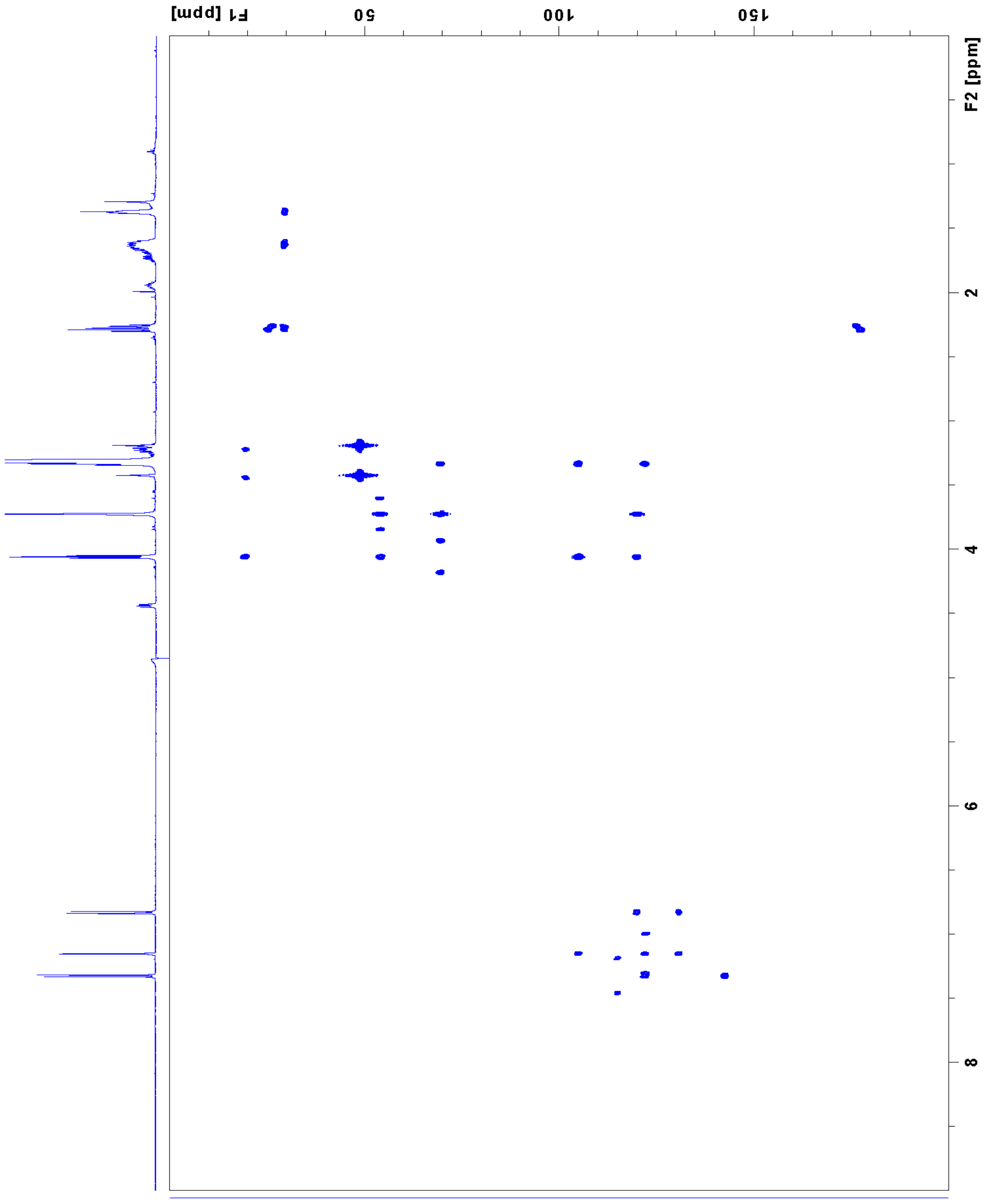

(**A**)

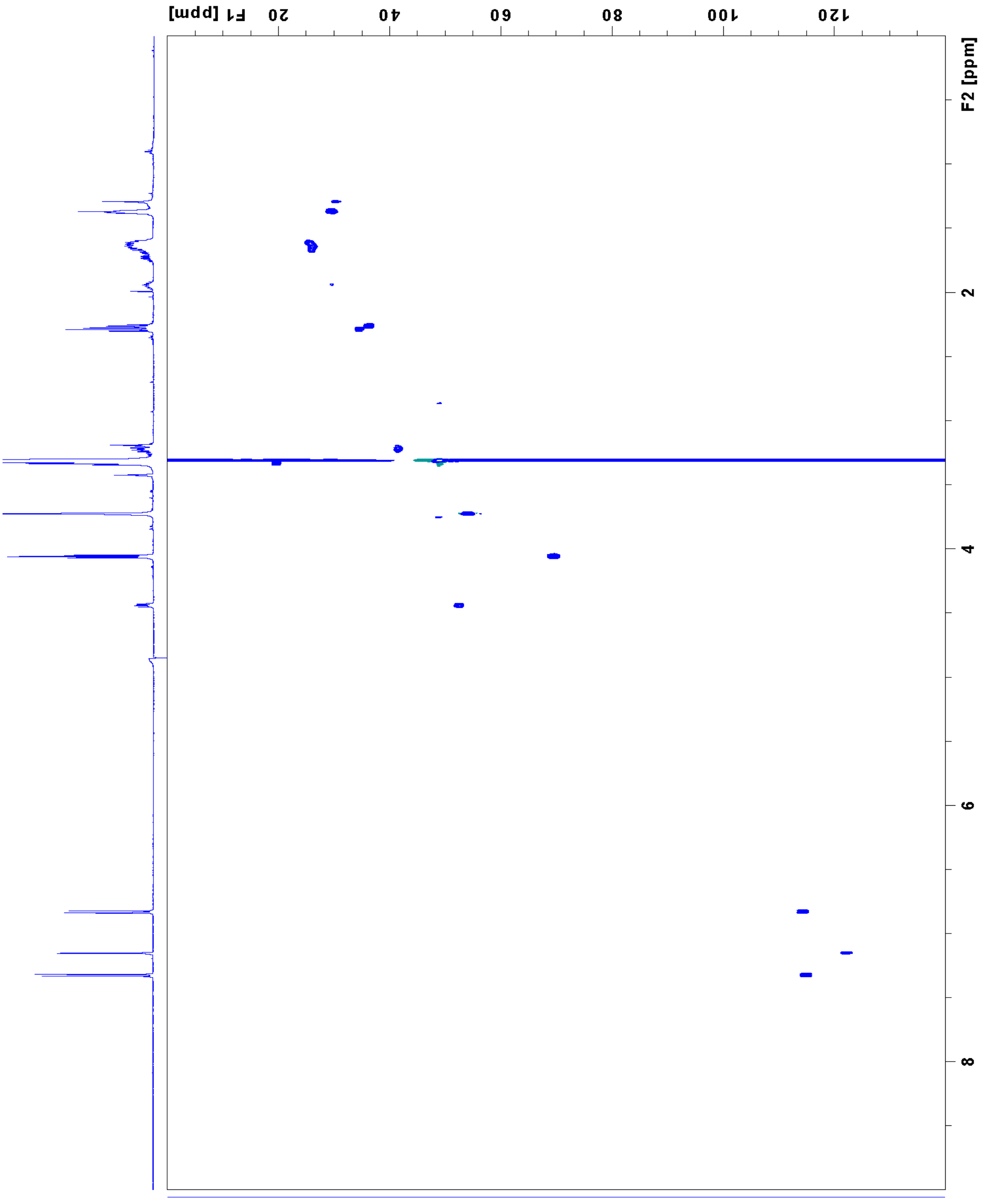

(**B**)

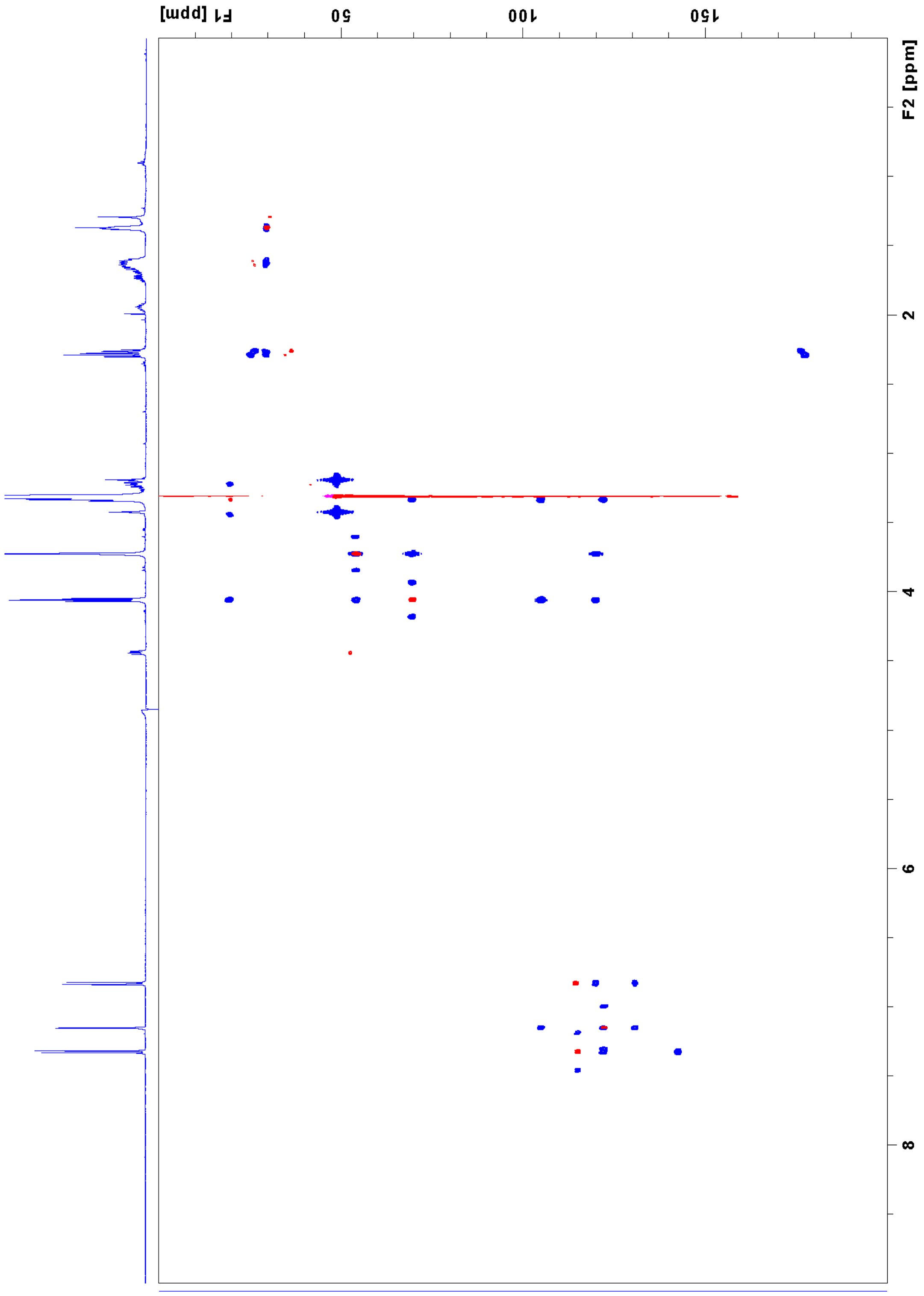

(**C**)

Figure S8. MS^2 spectrum, proposed structure, and proposed fragmentation scheme of the bioactive compound with *m/z* 401.235, eluting at 58.0 min. Most of the peaks in the MS^2 spectrum can be readily interpreted, e.g., in terms of water and CO losses, although it is generally unclear where exactly these losses occur.

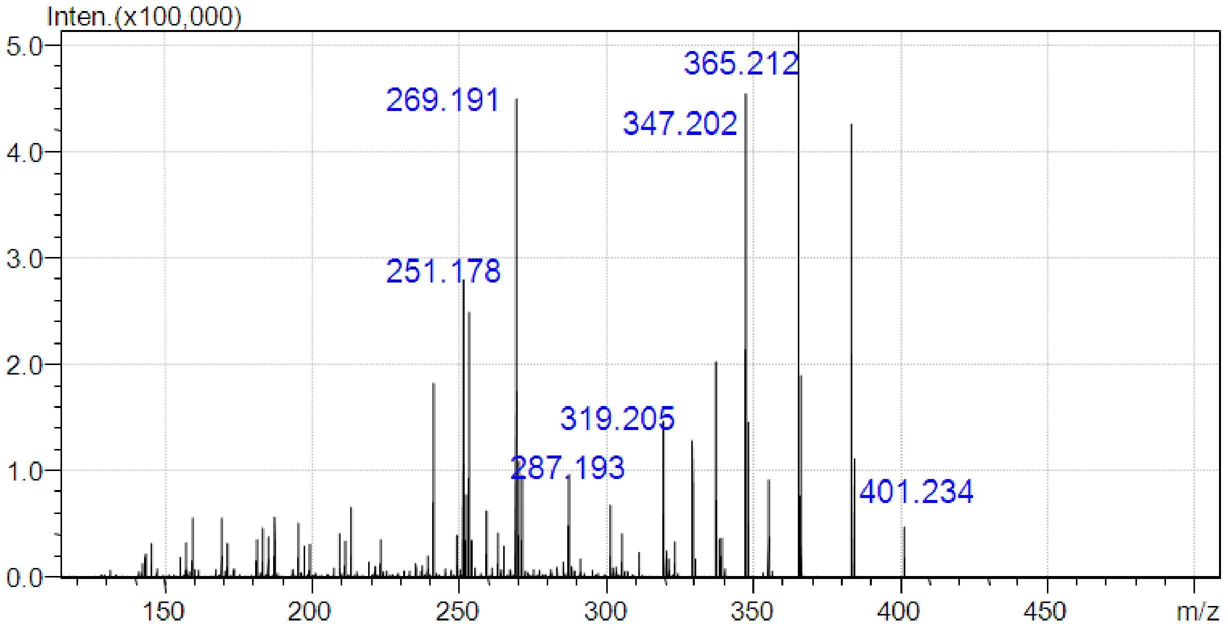

m/z	Formula	Interpretation
**401.234**	$C_{24}H_{33}O_5^+$	$[M+H]^+$
**383.222**	$C_{24}H_{31}O_4^+$	Loss of H_2O
**365.212**	$C_{24}H_{29}O_3^+$	Loss of 2 × H_2O; Loss of H_2O from *m/z* 383
355.228	$C_{23}H_{31}O_5^+$	Loss of HCOOH or CO and H_2O; Loss of CO from *m/z* 383
**347.202**	$C_{24}H_{27}O_2^+$	Loss of 3 × H_2O; Loss of H_2O from *m/z* 365
**337.218**	$C_{23}H_{29}O_2^+$	Loss of 2 × H_2O and CO; Loss of H_2O from *m/z* 355
329.191	$C_{24}H_{25}O^+$	Loss of 4 × H_2O; Loss of H_2O from *m/z* 347
319.205	$C_{23}H_{27}O^+$	Loss of 3 × H_2O and CO; Loss of H_2O from *m/z* 337
301.195	$C_{23}H_{25}^+$	Loss of 4 × H_2O and CO; Loss of H_2O from *m/z* 319
287.193		No matches within 5 mDa
**269.191**	$C_{19}H_{25}O^+$	Loss of $C_5H_8O_4$; Loss of $C_5H_4O_2$ from *m/z* 365
253.196	$C_{19}H_{25}^+$	Loss of $C_4H_4O_2$ from *m/z* 337
**251.178**	$C_{19}H_{23}^+$	Loss of H_2O from *m/z* 269
241.159	$C_{17}H_{21}O^+$	Loss of C_2H_4 from *m/z* 269
213.164	$C_{16}H_{21}^+$	Loss of CO from *m/z* 241

Figure S9. (**A**) COSY-NMR spectrum of the bioactive compound with *m/z* 401.235, eluting at 58.0 min. (**B**) TOCSY-NMR spectrum of the bioactive compound with *m/z* 401.235, eluting at 58.0 min.

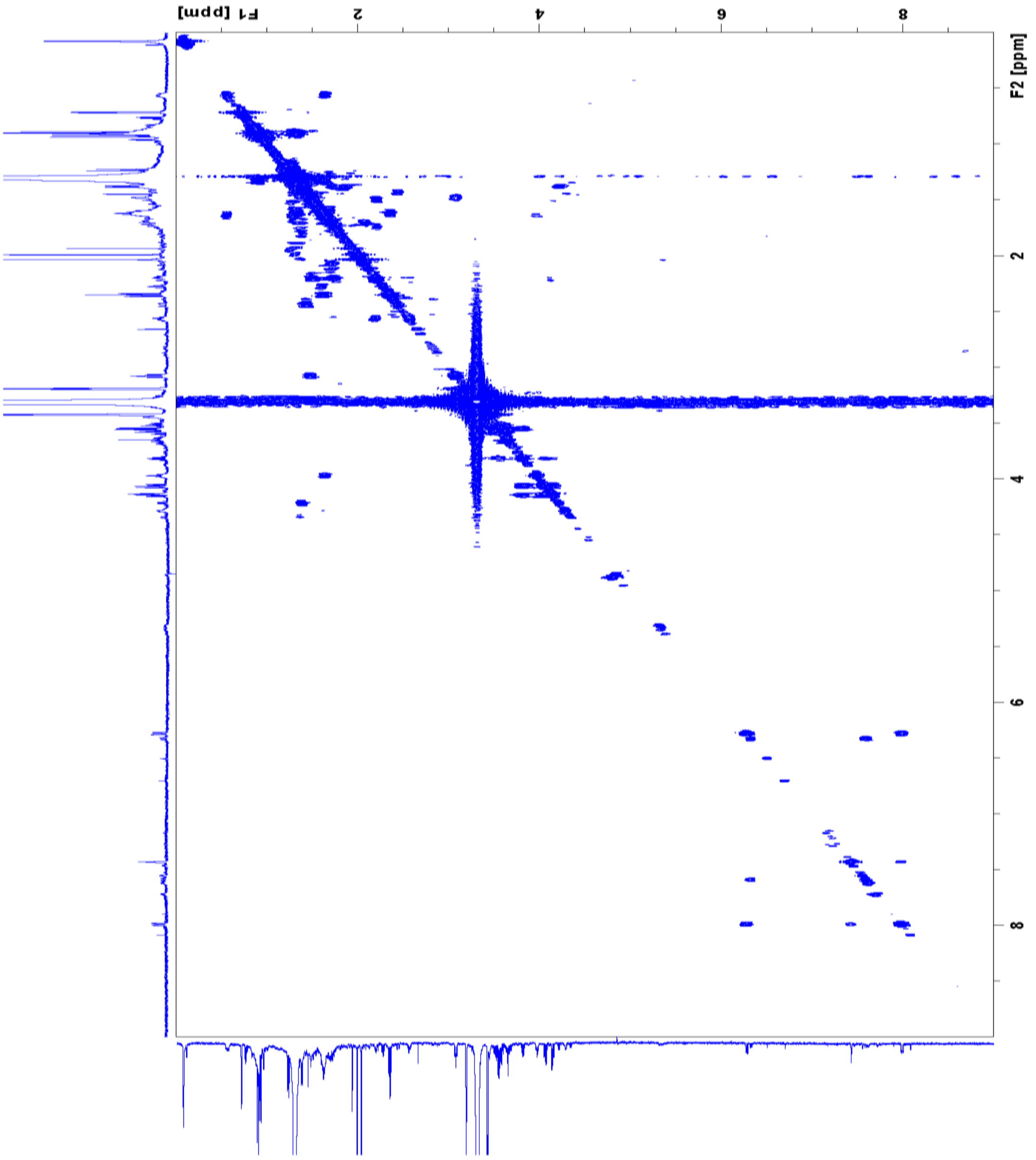

(**A**)

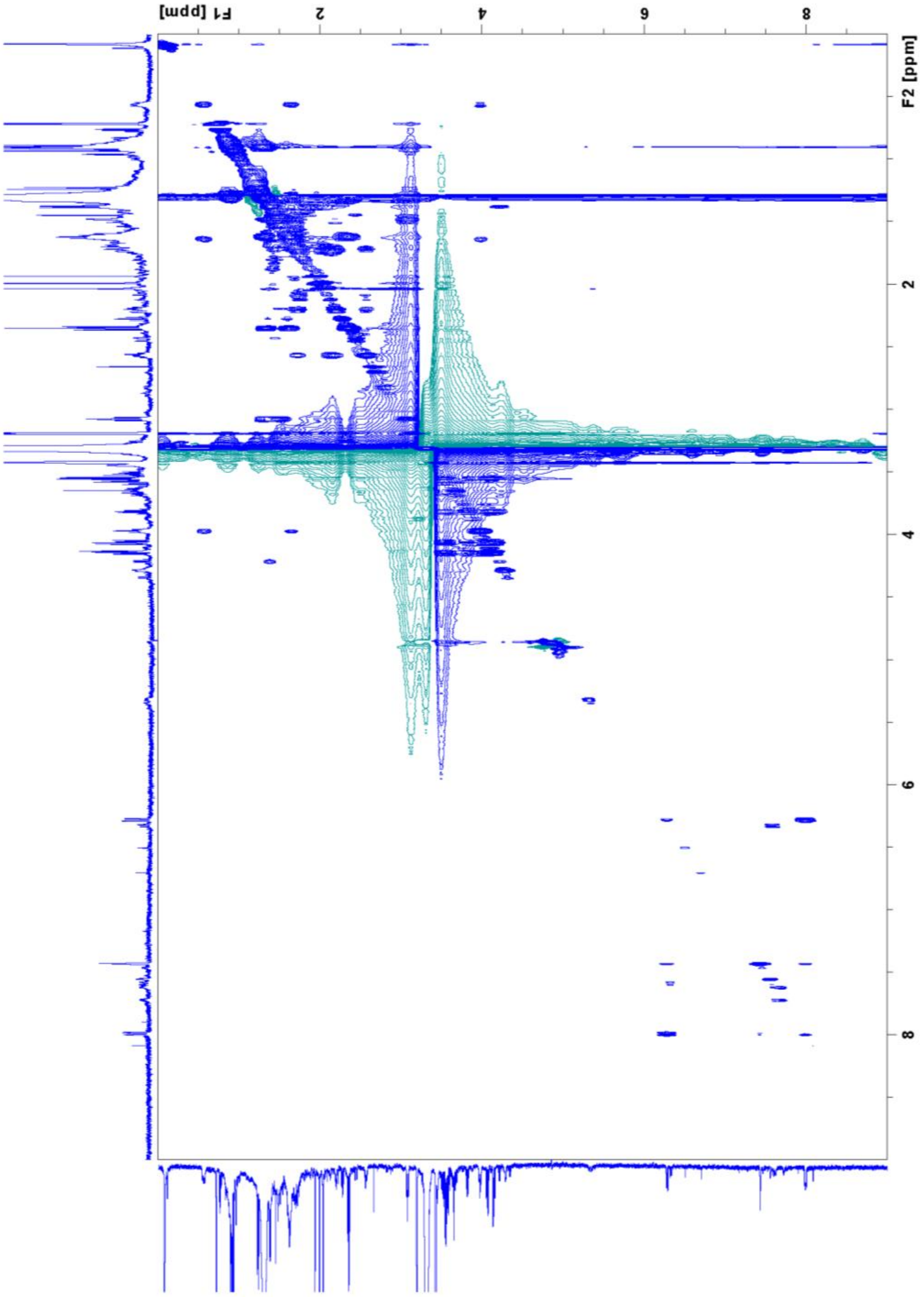

(B)

Figure S10. (**A**) MS^2 spectrum of the bioactive compound with *m/z* 713.412, eluting at 62.0 min. (**B**) Proposed structure and proposed fragmentation scheme of the bioactive compound with *m/z* 713.412, eluting at 62.0 min. The minor fragments can be considered as secondary fragments of the ion with *m/z* 331 and are consistent with subsequent losses of H_2O, CO and/or NH_3, e.g., loss of water to the ion with *m/z* 313, loss of NH_3 to *m/z* 296, loss of H_2O to *m/z* 278, and either loss of H_2O to *m/z* 260 or CO to *m/z* 250. The ion with *m/z* 253 is consistent with the loss of HN=C=CH and 2 × H_2O from the fragment ion with *m/z* 331.

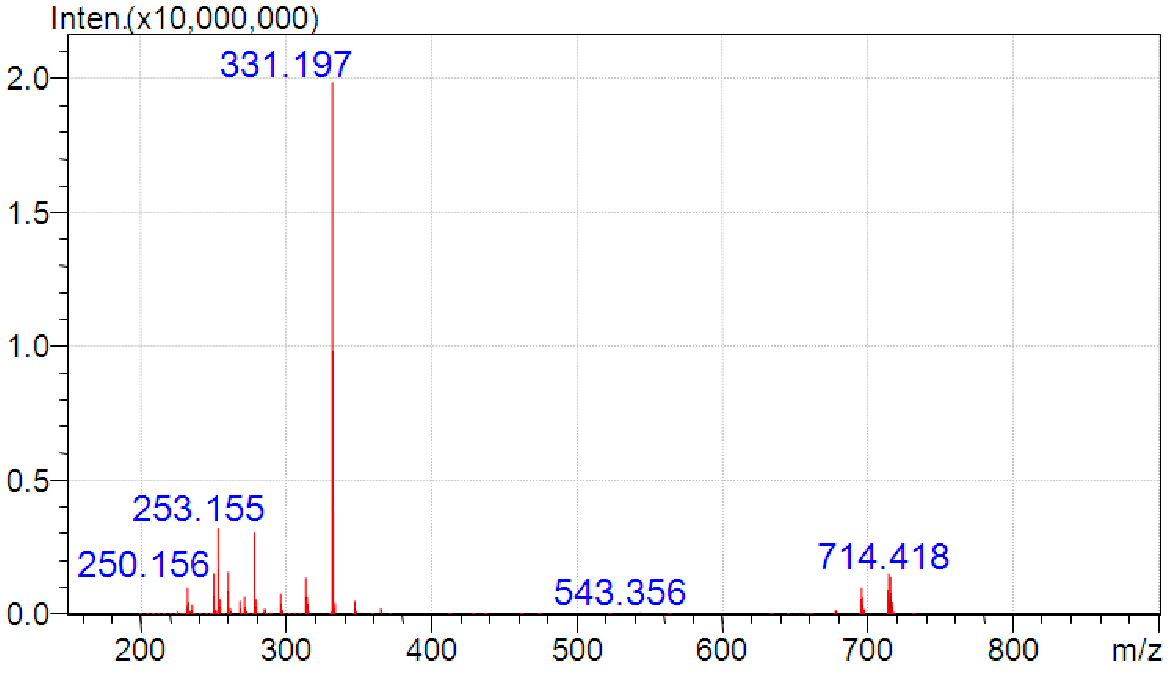

(**A**)

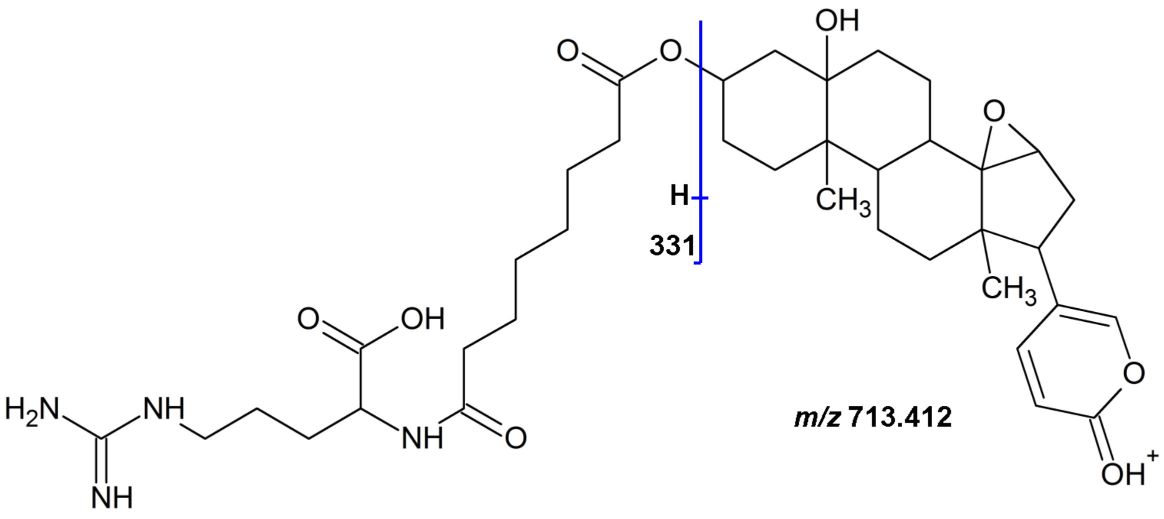

(**B**)
